# The effectiveness of the sarcopenia index in predicting septic shock and death in elderly patients with community-acquired pneumonia

**DOI:** 10.1186/s12877-022-03029-z

**Published:** 2022-04-19

**Authors:** Sha Huang, Lingdan Zhao, Zhaoyuan Liu, Yang Li, Xi Wang, Jianqun Li, Xiaoyan Chen

**Affiliations:** 1 Southwest Medical University Zigong Affiliated Hospital, Zigong, Sichuan Province China; 2 School of Nursing, Southwest Medical University, Luzhou, Sichuan Province China; 3 The Affiliated Suzhou Science and Technology Town Hospital of Nanjing Medical University, Jiangsu Province Suzhou, China

**Keywords:** Sarcopenia index, Serum creatinine, Serum cysteine C, Pneumonia, Septic shock, Mortality, Older adults

## Abstract

**Background:**

Community-acquired pneumonia (CAP) causes high morbidity and mortality in all age groups worldwide. Lower muscle radiodensity was associated with worse clinical outcomes (including shock) and higher in-hospital mortality. Prompt detection of sarcopenia in older adults with CAP is important. The measurement of muscle mass often involves specialized and expensive techniques. A relatively simple and inexpensive method such as the sarcopenia index (SI) to measure muscle mass would be helpful. Therefore, we performed a retrospective cohort study to assess the association between SI and septic shock risk and mortality in older patients with CAP.

**Study design:**

In this retrospective cohort study, information on hospitalized CAP patients, including general information and septic shock, were obtained from the medical record database of the Southwest Medical University Zigong Affiliated Hospital, China. Data on patient survival and mortality (all-cause) were acquired from government authorities and telephonic follow-up. Serum creatinine (Cr) and cystatin-C (CysC) levels on admission were included in the database. The SI was determined as the serum Cr/CysC ratio × 100 and the participants were assigned to low and high SI groups. The association between SI and septic shock was evaluated by logistic regression, and that between SI and mortality by Cox regression analysis.

**Results:**

In total, 769 older adults (≥ 60 years) with CAP were included, of which 480(62.4%) were male and 289(37.6%)were female. We found that the total prevalence of septic shock in older adults with CAP was 16.0%. In the female group, septic shock was more prevalent in the low SI group than in the high SI group (low SI vs. high SI, 22.22% vs. 11.52%, *p* = 0.024). Following adjustment for confounders, there was a significant association between high SI and a lower risk of septic shock in female patients (OR = 0.38, 95%CI: 0.16–0.94; *p* < 0.05). The total death toll of older adults with CAP was 332(43.2%). Irrespective of sex, there was a higher risk of mortality in the low SI group (total group: low SI vs. high SI, 63.02% vs. 36.57%, *p* < 0.001; male group: low SI vs. high SI, 63.03% vs. 39.34%, *p* < 0.001; female group: low SI vs. high SI, 73.61% vs. 28.57%, *p* < 0.001) and, after adjustment for confounding factors and irrespective of sex, high SI was a protective factor for mortality in older adults with CAP (total group: HR = 0.64, 95%CI: 0.48–0.84; *p* < 0.05; male: HR = 0.69, 95%CI: 0.49–0.97; *p* < 0.05; female: HR = 0.39, 95%CI: 0.24–0.62; *p* < 0.05).

**Conclusion:**

While the SI effectively predicts mortality in older adults with CAP, it was only found to be associated with septic shock in older females.

## Background

Community-acquired pneumonia (CAP) leads to high levels of morbidity and mortality among all ages throughout the world [1]. It is most prevalent in older adults, with the highest rates in people over the age of 80 years [2]. Mortality rates vary between 5 and 20% for patients hospitalized with CAP, reaching 50% in patients in intensive care [3]. Studies have pointed out that lower muscle radio-density is associated with poor clinical results (including shock) [4]. Sarcopenia is also associated with high in-hospital mortality in pneumonia patients [5]. Therefore, the timely identification of sarcopenia in older adults with CAP is important.

Sarcopenia has been defined as “a geriatric syndrome characterized by low muscle mass and inadequate mass strength and/or physical performance” [6]. The measurement of muscle mass usually involves specialized and expensive techniques, including dual-energy x-ray absorptiometry (DXA), bioelectrical impedance analysis (BIA), computer tomography (CT), and magnetic resonance imaging(MRI). It would thus be helpful to devise a relatively simple and inexpensive method for measuring muscle mass.

Recently, the use of the term “sarcopenia index” (SI) has been proposed in place of sarcopenia [7–10]. The SI is determined as the ratio between serum creatinine (Cr) and cystatin-C (CysC) and was defined by Kianoush et al. [9] as Cr/CysC × 100. The SI has been used effectively to measure both muscle mass and strength in different populations [8, 10–12]. Lower SI values indicate reduced muscle mass and were found to be effective in assessing the severity of malnutrition as well as predicting the need for intensive care treatment and mortality in critically ill patients [10].

Therefore, collecting the SI of older CAP patients at admission may provide insights into their prognosis that cannot be obtained by assessing the severity of pneumonia alone, making this assessment well-suited for improving patient-centred care settings 's recovery. Here, we conducted a retrospective cohort study to assess the association between SI and risk of septic shock and mortality in older CAP patients, assuming that low SI on admission is associated with higher septic shock and mortality in this patient population.

## Method

### Research design and patients

This was a single-institution retrospective observational study done at the Southwest Medical University Zigong Affiliated Hospital, China, between January 2016 and March 2021. Hospitalized CAP patients aged 60 and over the age of 60 were included. The exclusion criteria were estimated glomerular filtration rates (eGFR) below 30 ml/min/1.73 m^2^, renal replacement therapy, acute kidney injury, septic shock was present on admission and patients who refused clinical follow-up. The study was approved by the Research Ethics Committee of Southwest Medical University Zigong Affiliated Hospital (No.2021-06-01).

### Data collection

The patients’ clinical information, including age, sex, history of drinking and smoking, height, weight, chronic diseases(including hypertension, diabetes, coronary heart disease [CHD], and chronic obstructive pulmonary disease [COPD]), septic shock, and all-cause mortality, as well as blood test results after admission, were collected. The diagnosis of septic shock refers to the diagnostic criteria described by Font et al. in 2020 [13].

### Sarcopenia index

Fasting venous blood (after overnight fasting) was drawn by experienced geriatric nurses. The SI was calculated as serum Cr/CysC × 100. The patients were then divided into two groups according to the lowest quartile SI value: patients with scores in the lowest quartile were placed in the “low SI” group and the remainder assigned to the “high SI” group.

### Statistical analysis

SPSS 23.0 (IBM Corp. Armonk, NY, USA) was used for data analysis. Continuous data were expressed as means ± standard deviation (SD) or medians and interquartile ranges (IQR), based on the normality of distribution. Categorical data were represented as numbers (percentages). Patients’ baseline characteristics were compared using the Student's t test, Pearson’s χ2 test, and the logistic regression analysis was used to determine the associations between SI and septic shock and Cox regression analysis was used to determine the associations between SI and mortality. *P* < 0. 05 was considered to represent statistical significance.

## Results

We included 769 older adults with CAP, which of 480 (62.4%) were male and 289 (37.6%) were female. We divided the patients, including both male and female patients, into two groups according to their SI scores. In the low SI group: total < 51.08, male < 56.18, female < 44.92, and in the high SI group: total ≥ 51.08, male ≥ 56.18, female ≥ 44.92. In the total population, there were significant differences in age, serum albumin (ALB) level, eGFR, smoking history, drinking history, diabetes, and COPD between the low and high SI groups, while no differences were observed for BMI, hypertension, diabetes, and CHD. There were significant differences in male patients in the groups in terms of age, ALB level, eGFR, and smoking history, while females differed in age, ALB level, BMI, eGFR, drinking history, diabetes, and CHD between the low and high SI groups (Table 1).

**Table 1:** Baseline characteristics of participants according to the SI

Characteristics	Total *n* = 769			Male *n* = 480			Female *n* = 289		
	**low SI**	**high SI**	**p**	**low SI**	**high SI**	**p**	**low SI**	**high SI**	**p**
**Age years,mean(SD)**	82.08(8.94)	76.93(9.38)	< 0.001	79.24(9.91)	76.4(9.5)	0.005	82.66(8.24)	77.31(9)	< 0.001
**ALB level, g/l, mean(SD)**	32.81(6.1)	37.6(5.72)	< 0.001	32.78(5.84)	37.31(5.62)	< 0.001	34.88(6.73)	38.76(5.48)	< 0.001
** < 35 g/l,n(%)**	119(62.96)	162(28.32)		75(63.56)	103(28.85)		67(56.78)	36(21.43)	
** ≥ 35 g/,n(%)**	70(37.04)	410(71.68)		43(36.44)	254(71.15)		51(43.22)	132(78.57)	
**BMI, kg/m2, mean(SD)**	21.52(2.43)	21.82(3.05)	0.204	21.64(2.82)	21.66(3.01)	0.934	21.5(2.5)	22.33(3.08)	0.011
**eGFR, mL/min/1.73 m2,mean(SD)**	125.61(63.42)	85.16(31.17)	< 0.001	135.75(67.06)	84.95(29.3)	< 0.001	107(46.53)	74.92(25.66)	< 0.001
**Smoking history,n(%)**			< 0.001			0.002			0.85
no	158(82.29)	314(54.42)		62(52.1)	130(36.01)		70(97.22)	210(96.77)	
yes	34(17.71)	263(45.58)		57(47.9)	231(63.99)		2(2.78)	7(3.23)	
**Drinking history,n(%)**			< 0.001			0.594			0.046
no	165(85.94)	409(71.01)		75(63.03)	217(60.28)		68(94.44)	214(98.62)	
yes	27(14.06)	167(28.99)		44(36.97)	143(39.72)		4(5.56)	3(1.38)	
**Hypertension,n(%)**			0.569			0.762			0.697
no	112(58.33)	350(60.66)		69(57.98)	215(59.56)		57(79.17)	167(76.96)	
yes	80(41.67)	227(39.34)		50(42.02)	146(40.44)		15(20.83)	50(23.04)	
**Diabetes,n(%)**			0.02			0.073			0.04
no	144(75)	477(82.67)		92(77.31)	305(84.49)		37(51.39)	141(64.98)	
yes	48(25)	100(17.33)		27(22.69)	56(15.51)		35(48.61)	76(35.02)	
**CHD,n(%)**			0.238			0.256			0.024
no	125(65.1)	402(69.67)		82(68.91)	268(74.24)		36(50)	141(64.98)	
yes	67(34.9)	175(30.33)		37(31.09)	93(25.76)		36(50)	76(35.02)	
**COPD, n(%)**			0.001			0.081			0.734
no	164(85.42)	426(73.83)		89(74.79)	239(66.2)		66(91.67)	196(90.32)	
yes	28(14.58)	151(26.17)		30(25.21)	122(33.8)		6(8.33)	21(9.68)	

*SI sarcopenia index, ALB* serum albumin, *CHD* coronary heart disease, *COPD* chronic obstructive pulmonary disease

We found that the total prevalence of septic shock in older adults with CAP was 16.0%, among which the prevalence of septic shock in older male patients with CAP was 17.1% and that in older female patients was 14.2%. Septic shock was more prevalent among female patients with low SI scores (low SI vs. high SI, 22.22% vs. 11.52%, *p* = 0.024), but this was not apparent in either male patients or the overall group (Table 2). In order to explore the relationship between SI and septic shock, we used the low SI group as the control group. Model 1 showed that SI was associated with septic shock in older female adults with CAP (OR = 0.46, 95%CI: 0.23-0.91; *p* < 0.05). Adjustment for age, sex, smoking history, drinking history, BMI, eGFR, ALB level, and chronic disease (hypertension, diabetes, CHD, and COPD) (Model 2) resulted in an association between high SI and a lower risk of septic shock in older female patients with CAP (OR = 0.38, 95%CI: 0.16-0.94; *p* < 0.05) (Table 3).

**Table 2:** Differences in the distribution of septic shock and mortality between patients with low SI and high SI

Characteristics	Total *n* = 769			Male *n* = 480			Female *n* = 289		
	**Low SI**	**High SI**	**p**	**Low SI**	**High SI**	**p**	**Low SI**	**High SI**	**p**
**septic shock,n(%)**			0.153			0.19			0.024
no	155(80.73)	491(85.1)		94(78.99)	304(84.21)		56(77.78)	192(88.48)	
yes	37(19.27)	86(14.9)		25(21.01)	57(15.79)		16(22.22)	25(11.52)	
**mortality,n(%)**			< 0.001			< 0.001			< 0.001
no	71(36.98)	366(63.43)		44(36.97)	219(60.66)		19(26.39)	155(71.43)	
yes	121(63.02)	211(36.57)		75(63.03)	142(39.34)		53(73.61)	62(28.57)

**Table 3:** Correlations between SI and spetic shock

Adverse reactions	Total		Male		Female	
	**Model 1**	**Model 2**	**Model 1**	**Model 2**	**Model 1**	**Model 2**
Spetic shock						
low SI (ref)	1	1	1	1	1	1
high SI	0.73(0.48–1.12)	0.91(0.53–1.59)	0.71(0.42–1.19)	1.12(0.57–2.19)	0.46(0.23–0.91)*	0.38(0.16–0.94)*

Model 1: a non-adjusted model

Model 2: adjusting for age, gender, smoking history, drinking history, BMI, eGFR, ALB level, and disease (hypertension, diabetes, CHD, COPD)

^*^P < 0.05

We followed up the death outcomes of all patients, starting from July 1, 2021, and ending on July 7, 2021, for a total of 1 week. The total death toll of the older adults with CAP was 332(43.2%), of which 217(45.2%) were among older male patients with CAP, and 115(39.8%) were among the older female group. A total of 297 patients died within one year after being discharged from the hospital, and 35 people died after one year. The mortality rate among patients with low SI was higher irrespective of group (total group, male, or female) (total group: low SI vs. high SI, 63.02% vs. 36.57%, *p* < 0.001; male group: low SI vs. high SI, 63.03% vs. 39.34%, *p* < 0.001; female group: low SI vs. high SI, 73.61% vs. 28.57%, *p* < 0.001) (Table 2). Similarly, to explore the relationship between SI and mortality, we used the low SI group as the control group. In the uncorrected model (Model 1), SI was associated with death in the overall population of older adults with CAP, the male population, and the female population (total group: HR = 0.44, 95%CI: 0.35-0.55; *p* < 0.05; male group: HR = 0.46, 95%CI: 0.34-0.6; *p* < 0.05; female group: HR = 0.26, 95%CI: 0.18-0.37; *p* < 0.05). After adjusting for age, sex, smoking history, drinking history, BMI, eGFR, ALB level, and chronic disease (hypertension, diabetes, CHD, and COPD) (Model 2), we found that, regardless of group, high SI is a protective factor for mortality in older adults with CAP (total group: HR = 0.64, 95%CI: 0.48–0.84; *p* < 0.05; male group: HR = 0.69, 95%CI: 0.49-0.97; *p* < 0.05; female group: HR = 0.39, 95%CI: 0.24-0.62; *p* < 0.05) (Table 4).

**Table 4:** Correlations between SI and mortality

Adverse reactions	Total		Male		Female	
	**Model 1**	**Model 2**	**Model 1**	**Model 2**	**Model 1**	**Model 2**
**mortality**						
low SI(ref)	1	1	1	1	1	1
high SI	0.44(0.35–0.55)*	0.64(0.48–0.84)*	0.46(0.34–0.6)*	0.69(0.49–0.97)*	0.26(0.18–0.37)*	0.39(0.24–0.62)*

Model 1: a non-adjusted model

Model 2: adjusting for age, gender, smoking history, drinking history, BMI, eGFR, ALB level, and disease (Hypertension, diabetes, CHD, COPD)

^*^P < 0.05

## Discussion

In this study, it was found that, regardless of group (total, male, or female), high SI was a protective factor for mortality in older adults with CAP. It is well known that acute diseases can cause inflammation and necrosis of tissues, leading to inflammatory reactions. This inflammatory response leads to the secretion of various factors, including cytokines and C-reactive protein, that, in turn, leads to reduced food consumption, weight loss, and diminished muscle function, promoting both malnutrition and sarcopenia in the elderly. This leads to a vicious circle of malnutrition and myocytopenia, resulting in further inflammation and repeated illnesses [14, 15].

We also observed an association between high SI and reduced risk of septic shock in older adults with CAP. It could be postulated that sarcopenia induces a prolonged catabolic state that reduces the body’s ability to respond adequately to inflammatory stimuli. Under these conditions, immune function may be reduced, leading to the development of septic shock in response to infection [16]. However, our results cannot be generalized to male patients, and the specific reasons for this need to be further verified. Men and women differ in their protein and fat body composition and metabolism, and there are no studies that have examined the effects of sex differences on mortality in older CAP patients. Sex differences in the composition, functioning, and susceptibility to fatigue of skeletal muscles are well documented [17, 18]. Although male and female hormones clearly play a role in this dimorphism, the actual mechanism is not yet fully understood [19]. Here, we found that the predictive value of SI for septic shock in older patients with CAP differed between males and females, suggesting that muscle exhaustion and muscle loss have different effects on disease progression and prognosis in male and female patients, and it may be necessary to develop prevention and treatment strategies for these pathological conditions based on an understanding these sex differences.

The availability of a reliable method for measuring CAP severity may assist the triage and management of patients by assisting clinical decision-making regarding conservative or more aggressive management. Thus, although there are many studies on the evaluation of CAP prognosis, each of these methods is insufficient [20–23]. This is the first time that the SI has been used to predict septic shock and mortality from CAP in older adults. These results indicate that the SI can be used as a surrogate marker for predicting septic shock in older females with CAP and mortality in older adults with CAP. A larger cohort investigation is, however, needed for confirmation.

### Limitation

There are several limitations to our study. First, at 769 patients, the sample size was relatively small, which, together with the retrospective observational study design, could have led to possible selection bias and affected the correction of possible confounding factors. A large cohort study may be needed to confirm our findings. Secondly, we did not use DXA or BIA to determine the actual muscle mass.

## Conclusion

The SI is an effective predictor of mortality in older adults with CAP. However, an association between the SI and septic shock was only observed in older female patients.

## Data Availability

The datasets generated and analyzed during the current study are not publicly available due to this is a database which has a lot of important information and we are applying some important projects based on this. But this data sets will be available 2 years later and is also available now from the corresponding author on a reasonable request.

## Abbreviations

CAP: Community-acquired pneumonia
SI: Sarcopenia index
DXA: Dual-energy x-ray absorptiometry
BIA: Bioelectrical impedance analyses
MRI: Magnetic resonance imaging
CT: Computed tomography
Cr: Serum creatinine
CysC: Cystatin-C
OR: Odd ratio
CI: Confidence interval
HR: Hazard ratio
eGFR: Estimated glomerular filtration rates
CHD: Coronary heart disease
COPD: Chronic obstructive pulmonary disease
SD: Standard deviation
IQR: Medians and interquartile ranges
ALB: Serum albumin
